# Age Influences the Prognosis of Anaplastic Thyroid Cancer Patients

**DOI:** 10.3389/fendo.2021.704596

**Published:** 2021-07-27

**Authors:** Na Kong, Qiqi Xu, Ziqin Zhang, Aimin Cui, Shen Tan, Nan Bai

**Affiliations:** General Surgery of Beijing Jishuitan Hospital, The Fourth Clinical Medical College of Peking University, Beijing, China

**Keywords:** anaplastic thyroid cancer, age at diagnosis, staging system, AJCC, SEER

## Abstract

**Background:**

The staging system for patients with anaplastic thyroid cancer (ATC) was updated in the 8^th^ edition of the American Joint Committee on Cancer Staging Manual. A cut-off age of 55 years was stipulated as a prognostic factor for differentiated thyroid cancer; however, age was not considered for ATC patients. To this end, this study investigated the relationship between age at diagnosis and prognosis of ATC patients.

**Methods:**

The clinical information on ATC patients was acquired from the Surveillance, Epidemiology, and End Results Program public database. Youden’s index and X-tile analyses were used to calculate the high-point age at diagnosis associated with prognosis. Cox proportional hazards models, Kaplan-Meier curves, and 1000-person-year were then used for verifying the accuracy of the high-point age.

**Results:**

After inclusion/exclusion criteria was applied, 586 patients were included in this study. The high-point age was determined to be 70 years by both the Youden’s index and X-tile plot methods. The hazard ratio was 1.662 (95% confidence interval [CI]: 1.321-2.092), indicating that there was an increased risk of poor prognosis for patients > 70 years of age. The cancer-specific mortality rates per 1000-person-years for patients ≤ and > 70 years-old were 949.980 (95% CI: 827.323-1090.822) and 1546.667 (95% CI: 1333.114-1794.428), respectively. P-values were < 0.001 for the results shown above.

**Conclusion:**

Our study found that age influenced the prognosis of ATC patients. Furthermore, we determined that the high-point age at diagnosis was 70 years and that > 70 years of age was associated with a poor prognosis. These results provide a useful addition to the staging manual and can improve the diagnosis, treatment strategies and prognosis of ATC patients.

## Introduction

Thyroid cancer is the most common endocrine malignancy, and the incidence, is increasing at an alarming rate, especially in women (1–3). According to statistics, thyroid cancer has the fourth highest incidence rate of all malignant cancers (2, 4). The majority of thyroid tumors are differentiated thyroid cancers (DTCs), such as papillary and follicular tumors, which exhibit a good prognosis with a 5-year survival rate of 98% (3, 5). However, anaplastic thyroid cancers (ATC), a small subset of thyroid tumors, consists of undifferentiated cells with a median survival rate of 5 months and a 1-year-survival rate of < 20%, and account for 40–50% of thyroid cancer-specific mortality (6).

In the clinic, ATC usually presents as a neck mass that blocks the function of the esophagus and trachea and presents with symptoms of dysphagia, dysphonia or hoarseness, stridor, and/or dyspnea (7, 8). A previous multivariate analysis reported that age, presence of acute symptoms, leukocytosis, large tumor, and distant metastasis are independent factors for its prognosis (9). Another study also reported, surgical methods, chemotherapy, and gross residual disease to be amongst these factors (10).

It is common for patient age at diagnosis to be used as a prognostic factor for thyroid cancer patients (11). For DTC, age is included in the American Joint Committee on Cancer (AJCC) staging system (12, 13). Furthermore, in the most recent 8^th^ edition, the cut-off point for age at diagnosis was increased from 45 to 55 years (14). Since a linear association between age and survival has been reported in previous studies, the high-point age at diagnosis is dispute (15).

The staging system was updated in the 8^th^ edition of the AJCC manual (14); however, the age at diagnosis was not considered in the staging system for ATC patients (13). Thus, this study investigated the relationship between age at diagnosis and the prognosis of ATC patients to propose an accurate high-point age at diagnosis. Our results may provide additional information for the staging manual that may be of great value in the diagnosis, treatment, and prognosis of ATC patients.

## Materials and Methods

### Patients and Database

Data were acquired from the open access, authoritative database from the Surveillance, Epidemiology, and End Results (SEER) Program, launched in 1973 by the United States Centers for Disease Control and Prevention and National Cancer Institute. The SEER database includes information on patients with endocrine, respiratory, digestive system, and other tumors, and covers approximately 34.6% of the population in the United States. The data used in this study were obtained from a public anonymized database and ethics committee approval and informed consent were not required.

We enrolled 1286 patients with ATC from 2004 to 2017 using the ICD-0-3 SEER site/histology validation code 8021/3. The information entered for each patient included patient identification; race; age at diagnosis; sex; year of diagnosis; tumor (T)-stage; lymph node (N)-stage; metastasis (M)-stage; AJCC 7^th^ edition staging; multifocality; tumor size; tumor extension; bone brain, liver, and lung metastasis; and surgical method. Moreover, we excluded patients who had missing information as follows: 1) patients whose AJCC staging information was missing (698 patients) and 2) patients whose months of survival were recorded as unknown (2 patients).

### Statistical Analysis

We assessed the association between prognosis (mortality) and the age at diagnosis with Cox proportional hazards models. The results of the Cox analysis were adjusted for sex; race; year of diagnosis; tumor size; extension; multifocality; TNM-stage; bone, brain, liver, and lung metastasis; and surgical method. The optimal cut-off age was determined using the Youden’s index, which integrates sensitivity and specificity information with a value that ranges from 0-1, and X-tile plots, a tool for biomarker assessment and outcome-based cut-point optimization. Finally, Kaplan-Meier curves, Cox proportional hazards models, and mortality per 1000-person-years were used to determine the significance of the cut-off age.

Frequencies, proportions, and mean values ± standard deviations were used to present variables, as appropriate. P < 0.05 was considered statistically significant. Statistical analyses were performed using SPSS, version 22.0 (IBM Corp., Armonk, NY, USA), Stata/SE version 15 (Stata Corp, College Station, TX, USA), GraphPad Prism version 7 (GraphPad Software Inc., La Jolla, CA, USA), or X-tile 3.6.1 (Robert L Camp, M.D., Ph.D., Yale University, USA).

## Results

### General Characteristics of Study Population

This study included 586 patients with ATC. **Table 1** shows the demographic data, clinical characteristics, and treatment methods for patients with ATC. The mean age of the 586 patients was 69.66 ± 11.64 years-old, with a range of 26-85 years. Furthermore, 286 patients were > 70 years. Compared with patients whose age was ≤ 70 or > 70 years, the approximate ratio was 1:1. The female to male ratio was approximately 3: 2.

**Table 1:** Demographics and clinical characteristics of 586 patients with anaplastic thyroid cancer.

Variable	N (%)
Gender	
Female	353 (60.24)
Male	233 (39.76)
Race	
White	456 (78.08)
Black	46 (7.88)
Other	82 (14.04)
Age at diagnosis (mean, ± SD)	69.66 (± 11.64)
Year of diagnosis	
2010-2013	368 (62.80)
2014-2015	218 (37.20)
Tumor size, mean (SD), mm	76.89 (± 100.79)
Number of tumor foci	
1	399 (79.32)
≥ 2	104 (20.68)
Extension	
No	73 (13.70)
Yes	459 (86.30)
T category	
T4a	102 (19.69)
T4b	416 (80.31)
N category	
N0	261 (44.54)
N1	325 (55.46)
M category	
M0	312 (53.24)
M1	274 (46.76)
Bone metastasis	
Yes	60 (10.77)
No	497 (89.23)
Brain metastasis	
Yes	21 (3.79)
No	533 (96.21)
Liver metastasis	
Yes	24 (4.32)
No	532 (95.68)
Lung metastasis	
Yes	208 (37.28)
No	350 (62.72)
Surgical procedure	
Biopsy	327 (57.09)
Lobectomy	90 (15.71)
Subtotal or near-total thyroidectomy	28 (4.89)
Total thyroidectomy	128 (22.34)

SD, standard deviation.

### The Effect of Age and the High Point

According to **Table 2**, after adjusting for the variables described above, the hazard ratio for prognosis and overall age at diagnosis was 1.022 (95% confidence interval [CI]: 1.012-1.032), P < 0.001.

**Table 2:** Adjusted Cox proportional hazards analyses of cancer-specific mortality for patients with anaplastic thyroid cancer.

Variable	HR	95%CI	P-value
Age at diagnosis	1.022	1.012-1.032	<0.001
Race White	Ref		
Black	1.486	0.958-2.305	0.0771
Other	1.175	0.851-1.623	0.327
Gender	0.961	0.756-1.222	0.746
Year of diagnosis	0.941	0.749-1.182	0.600
Tumor size	1.001	1.000-1.002	0.008
Extension	0.880	0.501-1.546	0.656
Number of tumor foci	1.110	0.838-1.470	0.466
T stage	1.409	0.871-2.280	0.163
N stage	1.078	0.841-1.381	0.554
M stage	1.094	0.713-1.676	0.682
Bone metastasis	0.877	0.570-1.349	0.549
Brain metastasis	0.977	0.488-1.955	0.946
Liver metastasis	1.389	0.799-2.413	0.244
Lung metastasis	1.818	1.193-2.773	0.005
Surgical procedure Biopsy	Ref		
Lobectomy	0.734	0.533-1.009	0.057
Subtotal or near-total thyroidectomy	0.660	0.398-1.094	0.107
Total thyroidectomy	0.445	0.333-0.594	<0.001

HR, hazard ratio; CI, confidence interval.

**Figure 1:**
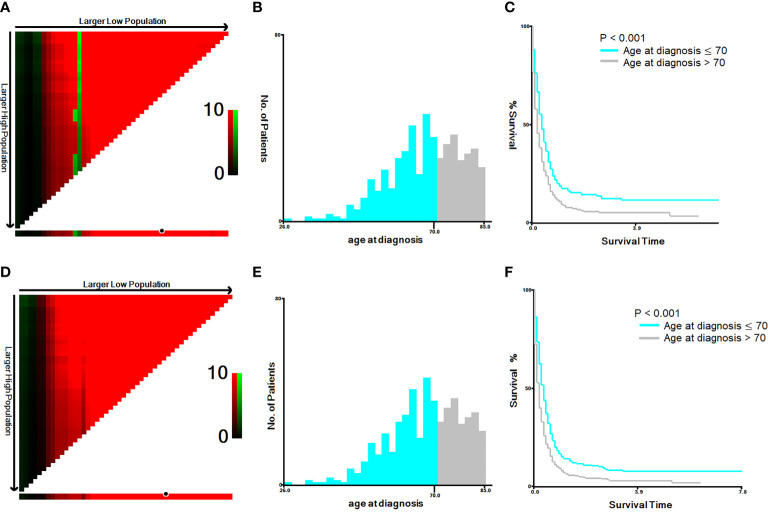
X-tile analysis of 586 anaplastic thyroid cancer patients based on cancer-specific mortality and overall mortality. ABBREVIATION: The training plots are shown in panel **(A, D),** with matched validation sets shown in panels **(B, E)** and **(C, F)**. The optimal cut-point highlighted by the black circle in panel **(A, D)** is shown on a histogram of the entire cohort **(B, E)** and a Kaplan-Meier plot **(C, F)**. The picture of **(A–C)** is for cancer-specific mortality; the picture **(D–F)** is for overall mortality. P values were determined by using the cut-point defined in the training set and applying it to the validation sets (P < 0.001).

The cut-off year for the age at diagnosis was determined to be 70 years old, using both the Youden’s index and X-tile plots with data on cancer-specific and overall mortality. Results of sensitivity and 1-specificity are shown in **Supplement Table 1**; the maximum value was 0.075, corresponding to 70 years. The results of X-tile plots are shown for cancer-specific mortality (**Figures 1A–C**) and overall mortality (**Figures 1D–F**) for patients under and over 70 years of age. The survival curves indicated that patients under 70 years of age had less cancer-specific and overall mortality than patients over 70 years, with Kaplan-Meier curves showing greater cancer-specific and overall mortality for patients > 70 years than for those ≤ 70 years of age (P < 0.001).

### Validation of Cut-Off Year

To confirm the accuracy of the cut-off age, we used Cox proportional hazards models with two groups, patients ≤ 70 years-old and > 70 years-old at diagnosis. After adjusting for the variables described above, the hazard ratio was 1.662 (95% CI: 1.321-2.092), with P < 0.001 (**Table 3**). The cancer-specific mortality rates were 949.980 (95% CI: 827.323-1090.822) and 1546.667 (95% CI: 1333.114-1794.428) 1000-person-years for patients who were diagnosed at ≤ 70 and > 70 years-old, respectively (**Table 4**). The overall mortality rates were 1082.316 (95% CI: 950.835-1231.978) and 1742.222 (95% CI: 1514.619-2004.027) 1000-person-years for the ≤ 70 years and > 70 years age groups, respectively (**Table 4**).

**Table 3:** Adjusted Cox proportional hazards analyses of cancer-specific mortality for patients with anaplastic thyroid cancer based on the cut-off age at diagnosis of 70 years.

Variable	HR	95%CI	P-value
Age at diagnosis ≤70	Ref		
>70	1.662	1.321-2.092	<0.001
Race White	Ref		
Black	1.481	0.950-2.307	0.083
Other	1.179	0.855-1.626	0.315
Gender	0.941	0.741-1.195	0.619
Year of diagnosis	1.662	1.321-2.092	<0.001
Tumor size	1.001	1.000-1.002	0.083
Extension	0.873	0.497-1.534	0.627
Number of tumor foci	1.130	0.853-1.497	0.394
T stage	1.402	0.886-2.269	0.169
N stage	1.070	0.836-1.370	0.592
M stage	1.151	0.751-1.764	0.517
Bone metastasis	0.911	0.594-1.398	0.669
Brain metastasis	0.861	0.430-1.722	0.672
Liver metastasis	1.330	0.767-2.309	0.310
Lung metastasis	1.735	1.138-2.644	0.010
Surgical procedure Biopsy	Ref		
Lobectomy	0.765	0.556-1.053	0.100
Subtotal or near-total thyroidectomy	0.619	0.370-1.035	0.068
Total thyroidectomy	0.424	0.318-0.566	<0.001

HR, hazard ratio; CI, confidence interval.

**Table 4:** Results of 1000-person-years for cancer-specific and overall mortality for anaplastic thyroid cancer patients with a dichotomous cut-off age at diagnosis of 70 years.

Variable		Fail	Rate	95% CI
Cancer-specific mortality	Age at diagnosis ≤ 70	201	949.980	827.323-1090.822
	Age at diagnosis > 70	174	1546.667	1333.114-1794.428
Overall mortality	Age at diagnosis ≤ 70	229	1082.316	950.835-1231.978
	Age at diagnosis > 70	196	1742.222	1514.619-2004.027

CI, confidence interval.

**Table 5** shows the comparisons of clinicopathological features between patients who were ≤ and > 70 years of age. Sex, multifocality, tumor size, N-stage, M-stage, bone metastasis, and overall mortality were significantly different between the two groups of patients; however, there were no differences for the other characteristics.

**Table 5:** Comparisons of clinicopathological characteristics between patients who were ≤ and > 70 years-old at the time of diagnosis of anaplastic thyroid cancer.

Variables		Age at diagnosis ≤ 70	Age at diagnosis > 70	P-value
Gender	Female	155 (43.91)	198 (56.09)	<0.001
	Male	145 (62.23)	88 (37.77)	
Race	White	235 (51.54)	221 (48.46)	0.235
	Black	28 (60.87)	18 (39.13)	
	Other	37 (45.12)	45 (54.88)	
Year of diagnosis	2010-2013	177 (48.10)	191 (51.90)	0.051
	2014-2015	123 (56.42)	95 (43.58)	
Tumor size, mean (± SD), mm		235 (± 361.168)	258.22 (± 377.791)	0.031
Number of tumor foci	1	205 (51.38)	194 (48.62)	0.006
	≥ 2	53 (50.96)	51 (49.04)	
Extension	No	37 (50.68)	36 (49.32)	0.563
	Yes	240 (52.29)	219 (47.71)	
T category	T4a	50 (49.02)	52 (50.98)	0.569
	T4b	217 (52.16)	199 (47.84)	
N category	N0	116 (44.44)	145 (55.56)	0.003
	N1	184 (56.62)	141 (43.38)	
M category	M0	147 (47.12)	165 (52.88)	0.035
	M1	153 (55.84)	121 (44.16)	
Bone metastasis	No	250 (50.30)	247 (49.70)	0.008
	Yes	41 (68.33)	19 (31.67)	
Brain metastasis	No	274 (51.41)	259 (48.59)	0.170
	Yes	14 (66.67)	7 (33.33)	
Liver metastasis	No	275 (51.69)	257 (48.31)	0.524
	Yes	14 (58.33)	10 (41.67)	
Lung metastasis	No	177 (50.57)	173 (49.43)	0.234
	Yes	116 (55.77)	92 (44.23)	
Surgical procedure	Biopsy	151 (46.18)	176 (53.82)	0.052
	Lobectomy	47 (52.22)	43 (47.78)	
	Subtotal or near-total thyroidectomy	17 (60.71)	11 (39.29)	
	Total thyroidectomy	76 (59.38)	52 (40.62)	
Survival month, mean (± SD)		8.46 (± 14.80)	4.72 (± 10.00)	<0.001
Cancer-specific mortality	Live	63 (57.27)	47 (42.73)	0.157
	Dead	237 (49.79)	239 (50.21)	
Overall mortality	Live	30 (73.17)	11 (26.83)	0.004
	Dead	270 (49.54)	275 (50.46)	

SD, standard deviation.

## Discussion

Although there is a relationship between age and prognosis for different types of malignancies, thyroid cancer is unique because age is included as a staging variable (16). In 1983, the 2^nd^ edition of the AJCC manual first settled on a dichotomous cut-off age of 45 years for DTC (17). The relationship between age and thyroid cancer has since been reevaluated. In 2018, a high-point age of 55 years was proposed because several recent studies suggested that mortality did not increase in patients with thyroid cancer before the age of 50 or 55 years at the time of diagnosis. The majority of deaths from thyroid cancer appeared to occur in those patients diagnosed after the age of 55 years (18, 19).

Besides patients diagnosed with DTC, the age at diagnosis should also be considered for other pathological types of thyroid cancer, such as ATC, for staging. Our study verified using Cox proportional hazard models that age influenced the prognosis of patients with ATC, and patients who were diagnosed at an older age had a worse prognosis. The Youden’s index and X-tile analyses showed that 70 years of age was the dichotomous cut-off for a worse prognosis for ATC patients.

It is reported that the largest group of patients with ATC were in their seventh and eighth decade of life (20). Although patients who were younger than 50 years-old have a better prognosis, such patients are few in number, and even rare under the age of 40 years (21). Thus, several studies have assessed the relationship between ATC severity and age at diagnosis and reported that age is an independent factor that was significantly associated with longer survival (9, 10).

Vladan’s et al. divided 150 ATC patients into 3 groups, <50 years-old, 51-70 years-old, and >70 years-old. They found that the chances of survival of the youngest age group were significantly better than that of the other two older age groups. In their study cohort, one-year survival of the youngest age group was observed in more than half the patients, whereas in the other two older groups one-year survival was 3-4 times less. Furthermore, during the first month from diagnosis of ATC, almost 30% of patients older than 70 years died, while less than 10% in the two younger age groups died (22).

Older patients were more likely to show a worse prognosis in thyroid cancer, which might have been affected by various factors. A study has pointed out that radioactive iodine (RAI), thyroid-stimulating hormone (TSH) levels, luteinizing hormone (LH) and follicle-stimulating hormone (FSH) homology, immune system decline, and genetic variation are associated with poor prognosis in thyroid cancer patients of advanced age (23).

ATC is a carcinoma with a high mortality rate. With increasing age, the selection of treatments for ATC patients is limited. In a previous study, researchers reported that subgroup analysis showed significance for radiotherapy, while multivariate analysis did not (7). In additional, they also pointed out that radiotherapy can provide benefit in terms of on loco-regional control but is not associated with an increase in survival (7).

For ATC patients, surgery had a greater impact on the overall survival (24). Furthermore, different surgical methods also impacted the survival rate. The prognosis of patients undergoing total thyroidectomy was significantly better than that of those undergoing partial thyroidectomy/lobectomy/subtotal thyroidectomy/subtotal thyroidectomy. The median survival time was also significantly improved in those who underwent total thyroidectomy (25). However, in addition to tumor size and location, the age of the patient also plays a crucial role in the choice of surgical method, and thus, the following adjuvant therapy.

There were limitations in this study. Because of the lack of serology-related indicators and molecular markers in the SEER database, we could not analyze the relationship between serology-related indicators and/or molecular markers and age at diagnosis of ATC. Furthermore, our study cohort had no limits of means to download the chemotherapy information, on external irradiation of ATC patients from the SEER database. Thus, we have not taken chemotherapy or external irradiation into consideration, which might influence the prognosis of patients. However, we will continue to pay attention to this issue in our future work. This study, evaluated cancer-specific and overall mortality in different age groups of ATC patients. Further, studies will be needed to investigate the associations between ATC prognosis and serology-related indicators and/or molecular markers.

In conclusion, we confirmed that the age at diagnosis influences ATC patients’ prognosis and calculated that the high-point of age at diagnosis was 70 years-old. We believe that these data should be considered in the next edition of the AJCC Staging Manual, and will aid in accurately diagnosing patients with ATC, provide for more specific treatments, and improve the prognosis prediction of these patients. We hope that future studies will be conducted to confirm our results and further study the relationship between age and patients with ATC.

## Data Availability Statement

Publicly available datasets were analyzed in this study. This data can be found here: SEER database.

## Author Contributions

All authors contributed to the article and approved the submitted version. NK, QX, and NB collected the data, and assured the integrity and accuracy of these data. NK and AC performed the data analysis and interpretation. NK prepared the figures. NB, ST, and ZZ provided administrative support. NK wrote the first draft of the manuscript.

## Conflict of Interest

The authors declare that the research was conducted in the absence of any commercial or financial relationships that could be construed as a potential conflict of interest.

## Publisher’s Note

All claims expressed in this article are solely those of the authors and do not necessarily represent those of their affiliated organizations, or those of the publisher, the editors and the reviewers. Any product that may be evaluated in this article, or claim that may be made by its manufacturer, is not guaranteed or endorsed by the publisher.
