# Supplementary material for: Age Influences the Prognosis of Anaplastic Thyroid Cancer Patients
Source: Front Endocrinol (Lausanne). 2021 Jul 27;12:704596. doi: 10.3389/fendo.2021.704596 (PMC8353231; doi:10.3389/fendo.2021.704596)
Supplement: Supplementary file 1 [file Table_1.docx]

Supplement table 1. Results of sensitivity and 1-specificity for anaplastic thyroid cancer patients based on cancer-specific and overall mortality

| Age at diagnosis | OS | | | CSS | | |
| --- | --- | --- | --- | --- | --- | --- |
|  | sensitivity | 1-specificity | Youden’s index | sensitivity | 1-specificity | Youden’s index |
| 25.000 | 1.000 | 1.000 | 0.000 | 1.000 | 1.000 | 0 |
| 29.500 | 0.998 | 1.000 | -0.002 | 0.998 | 1.000 | -0.002 |
| 34.500 | 0.994 | 1.000 | -0.006 | 0.994 | 1.000 | -0.006 |
| 36.500 | 0.993 | 1.000 | -0.008 | 0.992 | 1.000 | -0.008 |
| 38.000 | 0.991 | 1.000 | -0.011 | 0.989 | 1.000 | -0.011 |
| 39.500 | 0.989 | 0.976 | -0.004 | 0.987 | 0.991 | -0.004 |
| 40.500 | 0.987 | 0.976 | -0.006 | 0.985 | 0.991 | -0.006 |
| 42.000 | 0.985 | 0.951 | 0.001 | 0.983 | 0.982 | 0.001 |
| 44.000 | 0.983 | 0.951 | 0.010 | 0.983 | 0.973 | 0.01 |
| 45.500 | 0.978 | 0.927 | 0.013 | 0.977 | 0.964 | 0.013 |
| 46.500 | 0.972 | 0.927 | 0.018 | 0.973 | 0.955 | 0.018 |
| 47.500 | 0.969 | 0.927 | 0.014 | 0.968 | 0.955 | 0.013 |
| 48.500 | 0.963 | 0.927 | 0.008 | 0.962 | 0.955 | 0.007 |
| 49.500 | 0.954 | 0.927 | 0.008 | 0.954 | 0.945 | 0.009 |
| 50.500 | 0.945 | 0.927 | -0.002 | 0.943 | 0.945 | -0.002 |
| 51.500 | 0.932 | 0.927 | -0.017 | 0.929 | 0.945 | -0.016 |
| 52.500 | 0.917 | 0.854 | 0.016 | 0.916 | 0.900 | 0.016 |
| 53.500 | 0.910 | 0.829 | 0.017 | 0.908 | 0.891 | 0.017 |
| 54.500 | 0.897 | 0.805 | 0.011 | 0.893 | 0.882 | 0.011 |
| 55.500 | 0.875 | 0.780 | 0.040 | 0.876 | 0.836 | 0.04 |
| 56.500 | 0.861 | 0.756 | 0.043 | 0.861 | 0.818 | 0.043 |
| 57.500 | 0.851 | 0.732 | 0.042 | 0.851 | 0.809 | 0.042 |
| 58.500 | 0.839 | 0.707 | 0.036 | 0.836 | 0.800 | 0.036 |
| 59.500 | 0.822 | 0.659 | 0.047 | 0.819 | 0.773 | 0.046 |
| 60.500 | 0.796 | 0.610 | 0.035 | 0.790 | 0.755 | 0.035 |
| 61.500 | 0.780 | 0.585 | 0.037 | 0.773 | 0.736 | 0.037 |
| 62.500 | 0.750 | 0.488 | 0.039 | 0.739 | 0.700 | 0.039 |
| 63.500 | 0.710 | 0.488 | 0.027 | 0.700 | 0.673 | 0.027 |
| 64.500 | 0.679 | 0.439 | 0.021 | 0.666 | 0.645 | 0.021 |
| 65.500 | 0.662 | 0.415 | 0.022 | 0.649 | 0.627 | 0.022 |
| 66.500 | 0.648 | 0.366 | 0.046 | 0.637 | 0.591 | 0.046 |
| 67.500 | 0.607 | 0.366 | 0.044 | 0.599 | 0.555 | 0.044 |
| 68.500 | 0.563 | 0.366 | 0.050 | 0.559 | 0.509 | 0.05 |
| 69.500 | 0.527 | 0.366 | 0.052 | 0.525 | 0.473 | 0.052 |
| 70.500 | 0.505 | 0.268 | 0.075 | 0.502 | 0.427 | 0.075 |
| 71.500 | 0.486 | 0.268 | 0.065 | 0.483 | 0.418 | 0.065 |
| 72.500 | 0.459 | 0.220 | 0.074 | 0.456 | 0.382 | 0.074 |
| 73.500 | 0.428 | 0.220 | 0.050 | 0.422 | 0.373 | 0.049 |
| 74.500 | 0.400 | 0.195 | 0.050 | 0.395 | 0.345 | 0.05 |
| 75.500 | 0.374 | 0.171 | 0.052 | 0.370 | 0.318 | 0.052 |
| 76.500 | 0.336 | 0.146 | 0.072 | 0.336 | 0.264 | 0.072 |
| 77.500 | 0.316 | 0.122 | 0.070 | 0.315 | 0.245 | 0.07 |
| 78.500 | 0.290 | 0.122 | 0.063 | 0.290 | 0.227 | 0.063 |
| 79.500 | 0.268 | 0.098 | 0.058 | 0.267 | 0.209 | 0.058 |
| 80.500 | 0.239 | 0.098 | 0.035 | 0.235 | 0.200 | 0.035 |
| 81.500 | 0.218 | 0.098 | 0.023 | 0.214 | 0.191 | 0.023 |
| 82.500 | 0.183 | 0.073 | 0.015 | 0.179 | 0.164 | 0.015 |
| 83.500 | 0.167 | 0.073 | 0.018 | 0.164 | 0.145 | 0.019 |
| 84.500 | 0.143 | 0.049 | 0.034 | 0.143 | 0.109 | 0.034 |
| 86.000 | 0.000 | 0.000 | 0.000 | 0.000 | 0.000 | 0 |

Abbreviations: OS, overall survival; CSS, cancer-specific survival
